# Kinesiophobia and Pain Intensity Are Increased by a Greater Hallux Valgus Deformity Degree- Kinesiophobia and Pain Intensity in Hallux Valgus

**DOI:** 10.3390/ijerph17020626

**Published:** 2020-01-18

**Authors:** Patricia Palomo-López, Ricardo Becerro-de-Bengoa-Vallejo, Marta Elena Losa-Iglesias, Daniel López-López, David Rodríguez-Sanz, Carlos Romero-Morales, César Calvo-Lobo, Victoria Mazoteras-Pardo

**Affiliations:** 1 University Center of Plasencia, Universidad de Extremadura, 10600 Badajoz, Spain; patibiom@unex.es; 2 Facultad de Enfermería, Fisioterapia y Podología, Universidad Complutense de Madrid, 28040 Madrid, Spain; ribebeva@ucm.es (R.B.-d.-B.-V.); davidrodriguezsanz@ucm.es (D.R.-S.); vmazoter@ucm.es (V.M.-P.); 3 Faculty of Health Sciences, Universidad Rey Juan Carlos, 28922 Alcorcon, Spain; marta.losa@urjc.es; 4 Research, Health and Podiatry Group, Department of Health Sciences, Faculty of Nursing and Podiatry, Universidade da Coruña, 15403 Ferrol, Spain; daniellopez@udc.es; 5 Faculty of Sport Sciences, Universidad Europea de Madrid, Villaviciosa de Odón, 28670 Madrid, Spain; carlos.romero@universidadeuropea.es

**Keywords:** chronic pain, hallux valgus, musculoskeletal diseases, psychology

## Abstract

Background: Hallux valgus (HV) has been previously associated with psychological disorders. Thus, the purposes of this study were to associate kinesiophobia and pain intensity with HV deformity degrees, as well as predict kinesiophobia and pain intensity based on HV deformity and demographic features. Methods: A cross-sectional study was carried out recruiting 100 subjects, who were divided into HV deformity degrees, such as I-no HV (n = 25), II-mild (n = 25), III-moderate (n = 25), and IV-severe (n = 25) HV. Kinesiophobia total and domains (activity avoidance and harm) scores and levels were self-reported by the Tampa Scale of Kinesiophobia (TSK-11). Pain intensity was self-reported by the numeric rating scale (NRS). Results: Statistically significant differences (*p* < 0.01; η^2^ = 0.132–0.850) were shown for between-groups comparison of kinesiophobia total and domain scores (activity avoidance and harm) and levels, as well as pain intensity among HV deformity degrees. Post hoc comparisons showed statistically significant differences with a large effect size (*p* < 0.05; *d* = 0.85–4.41), showing higher kinesiophobia symptoms and levels and pain intensity associated with greater HV deformity degrees, especially for III-moderate and/or IV-severe HV deformity degrees versus I-no HV and/or II-mild deformity degrees. Both statistically significant prediction models (*p* < 0.05) for kinesiophobia (*R*^2^ = 0.300) and pain intensity (*R*^2^ = 0.815) were predicted by greater HV deformity degree and age. Conclusions: Greater kinesiophobia symptoms and levels and pain were associated with higher HV deformity degrees, especially severe and/or moderate HV with respect to no and/or mild HV. The kinesiophobia and pain intensity were predicted by greater HV deformity degree and age.

## 1. Introduction

The affectation of the toe body region may comprise up to 14% of the non-traumatic primary care consultations of the foot and ankle, being hallux valgus (HV) considered as one of the 10 most commonly documented non-traumatic conditions [1]. This condition may reach a prevalence of up to 23% in adults, showing an increase in female sex or higher age distribution [2]. HV may be defined as a complex deformity of the 1st metatarsophalangeal joint composed of great toe lateral drift and linked to joint subluxation [3]. Indeed, HV may impair quality of life related to foot health, increase depression, and alter muscle or connective tissue morphology of the plantar region, which seems to be linked to its degree of deformity [4,5,6,7].

Several psychological disorders, such as depression or sociability and vigor linked to general health-related quality of life alterations, have been associated with musculoskeletal conditions, which may increase with greater age ranges [8,9,10,11,12,13]. In addition, musculoskeletal disorders of the lower limbs may alter body stability, showing a greater instability in older adults [14,15,16]. Combining both factors, kinesiophobia and pain intensity seem to play a key role in musculoskeletal disorders prognosis [17,18,19]. Among these conditions, patellofemoral pain has been linked to greater higher kinesiophobia levels [20]. Currently, there is a lack of research studies detailing kinesiophobia and pain intensity in subjects suffering from HV deformity. Pain and kinesiophobia, defined as fear of movement under a painful condition [21,22], could be linked to a greater HV deformity degree as the higher HV deformity has been related to a worse foot health-related quality of life, greater depression, and presence of foot posture, pressure patterns, and function alterations [4,5,6,7,23,24].

Greater HV deformity degree has shown higher radiographic first metatarsophalangeal joint osteoarthritis severity in conjunction with physical and psychological conditions [4,5,6,7,23,24,25]. Thus, the purpose of this study was to find the association between kinesiophobia and pain intensity with HV deformity degrees. In addition, the secondary aim was to predict kinesiophobia and pain intensity based on HV deformity and demographic features. We hypothesized that higher kinesiophobia and pain intensity could be shown and predicted by a greater HV deformity degree.

## 2. Materials and Methods

### 2.1. Design

A cross-sectional study was performed according to the STrengthening the Reporting of OBservational studies in Epidemiology (STROBE) criteria [26]. Thus, kinesiophobia and pain intensity were compared under different HV deformity degrees. Furthermore, the ethics committee of Extremadura University (code: 175/2019) approved this research, and all subjects signed the informed consent form before the beginning of the study. Finally, the Helsinki declaration and all human experimentation rules were respected [27].

### 2.2. Sample Size Calculation

Kinesiophobia score was used as the main outcome measurement to carry out the sample size calculation because prior lower limb musculoskeletal conditions were linked to higher kinesiophobia symptoms [20]. Kinesiophobia total score assessed with the Spanish validated version of the Tampa Scale of Kinesiophobia–11 items (TSK-11) [21,22] of a pilot study (n = 40 subjects) with 4 groups of HV deformity degree (n; TSK-11 mean ± SD), divided into I–no HV (n = 10 participants; 21.70 ± 5.41 points), II–mild (n = 10; 20.70 ± 4.62 points), III–moderate (n = 10; 22.60 ± 3.83 points), and IV–severe (n = 10; 24.80 ± 4.18 points) HV deformity degree, was used for the sample size calculation by the one-way, omnibus, and fixed-effects analysis of variance (ANOVA) F test using G*Power 3.1.9.2 software version. Indeed, a partial Eta-squared (η^2^) of 0.109, an effect size of 0.349, an α error probability of 0.05, a number of 4 groups, and a power (1-β error probability) of 0.80 were used for this sample size calculation procedure. Thus, a total sample size of 96 subjects, 24 for each group, was calculated with an actual power of 0.813. Finally, a total sample size of 100 subjects, 25 for each group, was included in the present study.

### 2.3. Sample

A total sample of 100 subjects with different HV deformity degrees was recruited by a consecutive convenience sampling method in an outpatient clinic from March to November 2019 [4,5,6,7]. Inclusion criteria comprised subjects older than 18 years old, being healthy subjects for the control group classified as I degree–no HV presence (n = 25), as well as patients with HV deformity for cases groups, such as II degree–mild HV (n = 25), III degree–moderate HV (n = 25), and IV degree–severe HV (n = 25) [28,29].

Systemic diseases, neurological conditions, arthritis, neoplasm, autoimmune pathology, vascular alterations, neuropathic disorders or radiculopathies, sprains, fractures, tendinopathies, surgeries, presence of dysmetria with length difference greater than 1 cm between both lower extremities, mental disorders, or cognitive conditions were considered as exclusion criteria according to the medical record [7,30].

### 2.4. HV Deformity Degrees

A specialized podiatrist carried out the HV deformity degree diagnosis by the Manchester Scale [28]. This tool might be considered as a non-invasive technique in order to measure the HV deformity degree using a standardized photograph set, divided into I degree (no HV), II degree (mild HV), III degree (moderate HV), and IV degree (severe HV). This scale showed an excellent inter-examiner repeatability (κ = 0.86) [29]. Also, excellent inter-examiner reliability and validity were shown for the HV angle between photographic measures and radiographs. Intraclass correlation coefficients (ICCs > 0.96) and the Pearson’s correlation coefficient (*r* = 0.96) were categorized as excellent. Despite this method was recommended in order to avoid the cost and radiation exposure secondary to radiographs, foot radiographs might be considered as the current standard in clinical practice, and thus HV angle clinical measurements have been recommended if it is not possible or necessary to perform radiographs [29].

### 2.5. Demographic Data

Demographic data comprised age (years), sex (female or male), body mass index (kg/cm^2^) [31], height (cm), weight (kg), and pain chronicity, measured as duration in months with painful HV [7,30].

### 2.6. Outcome Measurements

Kinesiophobia total score was considered as the main outcome measurement and evaluated with the Spanish validated version of the Tampa Scale of Kinesiophobia–11 items (TSK-11) [21,22]. Secondary outcome measures were activity avoidance and harm domains scores of kinesiophobia, as well as fear of movement or kinesiophobia levels categorized by the TSK-11 score [21,22], and the pain intensity score measured by the numeric rating scale (NRS) [32,33]. According to these scales, both tools were self-reported by the study’s subjects.

#### 2.6.1. TSK-11

The Spanish validated version of the TSK-11 was self-reported by all study’s subjects in order to detail kinesiophobia symptoms total scores, activity avoidance and harm domains scores of kinesiophobia, and levels fear of movement or kinesiophobia [21,22]. Kinesiophobia was considered as an adaptive response to the threat, which might consequently generate maladaptive or avoidance behaviors with an increase of fear and/or pain as well as activities limitation and/or fear of movement [34,35,36]. Future disability of a musculoskeletal condition might be predicted by fear or movement or kinesiophobia [34]. This scale was composed of total kinesiophobia symptoms score and two domains, including activity avoidance and harm under kinesiophobia. This scale was scored using 4 points Likert-type scale, indicating higher scores as an increase of fear of pain, movement, or damage. In addition, TSK-11 total scores were categorized into kinesiophobia levels of fear of movement, including no fear of movement (0–17 points), slight fear of movement (18–24 points), moderate fear of movement (25–31 points), severe fear of movement (32–38 points), and maximum fear of movement (39–44 points) [21,22]. Adequate psychometric properties were reported for this scale, showing an internal consistency with Cronbach’s α of 0.78, test-retest with ICC of 0.82, standard error of measurement with SEM of 3.16, responsiveness of −1.19, minimum clinical important difference with MCID of 4.80, and minimum detectable change with MDC of 5.60 [21,22,37,38,39].

#### 2.6.2. NRS

Pain intensity was measured by the NRS. This tool showed 11 points ranged from 0 (no pain) to 10 (highest pain intensity) points. Subjects were asked to mark the subjective pain intensity of the painful HV (Hallux valgus) by a finger on the scale composed of a graphic representation with 11 spaces. This scale was stated as a valid and reliable scale to evaluate subjective pain intensity in adults and older adults [32,33]. High convergent validity (0.79–0.95) was shown with respect to the VAS (Visual Analogue Scale) [40]. The MDC and MCID were set at 2 points for lower limb musculoskeletal conditions [41,42,43].

### 2.7. Statistical Analysis

The software version 24th of the Statistical Package for Social Sciences (from IBM Corp; Armonk, NY, USA) was utilized to carry out all data analyses by an α error of 0.05, a *p*-value < 0.05 as statistically significant, and a 95% confidence interval (CI).

First, quantitative data analyses were performed by the Shapiro–Wilk test to determine normality distributions. Second, all data were described by the mean ± standard deviation (SD). Third, one-way analysis of variance completed with Bonferroni’s correction post hoc analyses was used to assess between-group differences for parametric data. Fourth, Kruskal–Wallis test completed with Bonferroni’s correction post hoc analyses were used to assess between-groups differences for non-parametric data. For outcome measurements, effect size was calculated by Eta-squared (η^2^) coefficients for comparisons among all groups, as well as Cohen’s *d* coefficients for comparisons between paired groups and categorized into very small effect size (*d* < 0.20), small effect size (*d* = 0.20–0.49), medium effect size (*d* = 0.50–0.79), and large effect size (*d* > 0.8) [44,45]. Finally, categorical data were described as frequency (n) and percentage (%). In addition, Chi-square tests were applied to assess differences among all groups.

Furthermore, multivariate predictive analyses were performed by means of two linear regression models. Both models were carried out by the stepwise selection method; as well as *R*^2^ coefficients were determined to show the quality of adjustment [46]. The 1st linear regression model included demographic data, pain intensity (NRS), chronicity, and HV deformity degree as independent variables, as well as kinesiophobia total score (TSK-11) as the dependent variable. The 2nd linear regression model included demographic data, kinesiophobia total score, levels of fear of movement of kinesiophobia, activity avoidance, and harm domains of kinesiophobia (TSK-11), and HV deformity degree as independent variables, as well as pain intensity (NRS) as the dependent variable. F probability pre-established parameters ranged from *p*_in_ = 0.05 to *p*_out_ = 0.10, and *p*-values < 0.05 for statistical significance with a 95% CI were considered for these analyses.

## 3. Results

### 3.1. Demographic Data

Statistically significant differences (*p* < 0.05) were shown for demographic data, except for weight (*p* = 0.608), showing that a higher HV deformity degree was associated with greater age, height, body mass index (BMI), and chronicity, as well as female sex (Table 1).

### 3.2. Kinesiophobia Total Score

Statistically significant differences (*p* < 0.001; η^2^ = 0.203) were shown for between-groups comparison of kinesiophobia total scores among HV deformity degrees by the one-way ANOVA test. Post hoc comparisons showed statistically significant differences with a large effect size (*p* < 0.05; *d* = 0.85–1.44), showing higher kinesiophobia symptoms for III-moderate and IV-severe HV deformity degrees with respect to I-no HV deformity degree, as well as for IV-severe HV deformity degree versus II-mild HV deformity degree. The rest of the post hoc comparisons did not show statistically significant differences (*p* < 0.05) (Table 2).

### 3.3. Activity Avoidance Score (TSK-11)

Statistically significant differences (*p* < 0.001; *η^2^* = 0.168) were shown for between-groups comparison for the activity avoidance domain of kinesiophobia among HV deformity degrees by the Kruskal–Wallis test. Post hoc comparisons showed statistically significant differences with a large effect size (*p* < 0.01; *d* = 0.86–1.34), showing higher activity avoidance kinesiophobia symptoms for IV-severe HV deformity degree with respect to I-no HV and II-mild HV deformity degrees. The rest of the post hoc comparisons did not show statistically significant differences (*p* < 0.05) (Table 2).

### 3.4. Harm Score (TSK-11)

Statistically significant differences (*p* = 0.002; *η^2^* = 0.132) were shown for between-groups comparison for the harm domain of kinesiophobia among HV deformity degrees by the Kruskal–Wallis test. Post hoc comparisons showed statistically significant differences with a large effect size (*p* = 0.001; *d* = 1.07), showing higher harm kinesiophobia symptoms for IV-severe HV deformity degree with respect to I-no HV deformity degree. The rest of the post hoc comparisons did not show statistically significant differences (*p* < 0.05) (Table 2).

### 3.5. Pain Intensity (NRS)

Statistically significant differences (*p* < 0.001; *η^2^* = 0.850) were shown for between-groups comparison for the pain intensity among HV deformity degrees by the Kruskal–Wallis test. Post hoc comparisons showed statistically significant differences with a large effect size (*p* < 0.001; *d* = 3.31–4.41), showing higher pain intensity for III-moderate and IV-severe HV deformity degree with respect to I-no HV and II-mild HV deformity degree. The rest of the post hoc comparisons did not show statistically significant differences (*p* < 0.05) (Table 2).

### 3.6. Kinesiophobia Levels of Fear of Movement (TSK-11)

**Figure 1:**
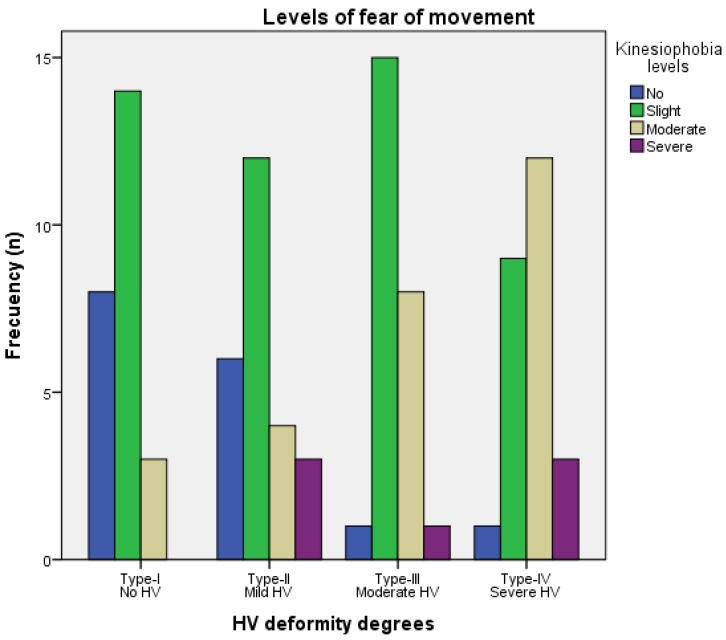
Bar graph showing the kinesiophobia levels of fear of movement (TSK-11), such as no fear (0–17 points), slight (18–24 points), moderate (25–31 points), severe (32–8 points), and maximum (39–4 points) kinesiophobia levels among different HV deformity degrees. Abbreviations: HV, hallux valgus; TSK-11, Tampa Scale of Kinesiophobia–11 items.

Statistically significant differences (*p* = 0.007; χ^2^ = 22.556) were shown for between-groups comparison for the kinesiophobia levels of fear of movement among HV deformity degrees by the Chi-squared test, showing higher kinesiophobia levels with greater HV deformity degree, especially for moderate kinesiophobia level (Figure 1).

### 3.7. Prediction Models

Kinesiophobia total score (TSK-11) showed one statistically significant prediction model (*R*^2^ = 0.300) based on age (R2 = 0.271; β = +0.101; F[1,98] = 35.978; P < 0.001) and HV deformity degrees (R2 = 0.029; β = +1.050; F[1,97] = 4.033; P = 0.047), predicting higher kinesiophobia total scores based on greater age and HV deformity degree. Therefore, this prediction model excluded the rest of independent variables (P > 0.05) as the kinesiophobia total score (dependent variable) was not predicted by sex, height, weight, BMI, chronicity, and pain intensity (independent variables) according to the pre-established parameters for F probability (Table 3).

Pain intensity (NRS) showed one statistically significant prediction model (*R*^2^ = 0.815) based on HV deformity degrees (*R*^2^ = 0.776; β = +0.276; F[1,98] = 339.076; P < 0.001) and age (R2 = 0.040; β = +1.050; F[1,97] = 20.782; P < 0.001), predicting higher pain intensity based on greater HV deformity degree and age. Therefore, this prediction model excluded the rest of independent variables (P > 0.05) as the pain intensity (dependent variable) was not predicted by sex, height, weight, BMI, kinesiophobia total scores, kinesiophobia activity avoidance and harm domain scores, and kinesiophobia levels of fear of movement (TSK-11) according to the pre-established parameters for F probability (Table 4).

## 4. Discussion

Despite higher HV deformity degree has been previously associated with psychological disorders [4,5,47], this study might be considered as the first cross-sectional study detailing greater kinesiophobia symptoms, total scores as activity avoidance and harm domains scores, as well as pain intensity associated with higher HV degree deformity, especially for severe and/or moderate HV deformity degrees with respect to non-presence of HV and/or mild HV deformity degrees. Indeed, moderate kinesiophobia level deformity showed a clear increase according to greater HV deformity degrees. Thus, pain and fear of movement under HV condition might be linked to III and IV deformity degrees compared to I and II deformity degrees according to prior studies associating greater HV deformity with worse quality of life related to foot health, higher depression, as well as foot posture, pressure patterns, and function alterations [4,5,6,7,23,24]. Our findings were in line with prior research studies detailing kinesiophobia and pain in different musculoskeletal conditions, such as patellofemoral pain [20], temporomandibular conditions [48], chronic fatigue syndrome and/or fibromyalgia [49], whiplash-associated conditions and/or low back pain [50], chronic mechanical neck pain [51], or migraine [52].

In addition, our study showed that age and the HV deformity degree were shown as predictors for kinesiophobia symptoms and pain intensity. These findings were in accordance with prior studies reporting that psychological disorders were linked to musculoskeletal conditions, increasing this association with greater age distribution [8,9,10,11,12,13]. In addition, greater instability was shown in older adults under musculoskeletal disorders [14,15,16]. Finally, higher HV deformity was associated with worse physical and psychological factors [4,5,6,7,23,24].

### 4.1. Future Studies 

Future studies should propose interventions in order to reduce kinesiophobia and pain intensity in patients with HV, such as myofascial pain interventions [53,54], neural mobilization techniques [55], or surgical procedures [47]. In addition, other outcome measurements should be evaluated in order to determine the influence of HV mechanical soft tissue properties on pain and kinesiophobia, such as myofascial trigger points evaluation [56], sonoelastography [57], pressure pain threshold [58], or thermography [59]. Finally and most importantly, an x-ray should be included in future studies as the gold stand, as well as ultrasound imaging could be used in advance to decline other possible pathologies [29].

### 4.2. Limitations

The following limitations could be acknowledged in this study. Firstly, socio-economic, civil, or working status should be considered for future studies. In spite of the pain intensity of HV was measured by the NRS [32,33], pain location, distribution, or type (neurological or musculoskeletal) were not collected. Second, despite the presented prediction models determined the influence of demographic data on our findings, future studies should detail the influence of age ranges on kinesiophobia and pain intensity according to our multivariate regression analyses. Thirdly, pregnant women could influence the psychological status and should be considered in future research studies [60]. Finally, despite exclusion criteria were considered according to the medical record, imaging examination was not performed (i.e., ultrasound and/or x-ray) to rollout other underlying pathologies (i.e., Morton neuroma, stress fracture, metatarsal bursitis, and others), which should be included in future studies. In addition, foot x-ray might provide additional information about the other metatarsal angles, sesamoid displacement, or underlying pathologies [29].

## 5. Conclusions

Greater kinesiophobia symptoms and levels and pain were associated with higher HV deformity degrees, especially severe and/or moderate HV with respect to no and/or mild HV. The kinesiophobia and pain intensity were predicted by greater HV deformity degree and age.

## Figures and Tables

**Table 1:** Demographic data among different HV deformity degrees.

Sociodemographic Characteristics		No HV Degree-I (n = 25)	Mild HV Degree-II (n = 25)	Moderate HV Degree-III (n = 25)	Severe HV Degree-IV (n = 25)	*p*-Value
Age (years)		37.52 ± 15.19	37.16 ± 19.53	59.24 ± 18.35	68.40 ± 14.60	<0.001 †
Weight (kg)		75.80 ± 16.12	70.80 ± 15.03	72.92 ± 10.57	73.08 ± 13.03	0.608 †
Height (m)		1.70 ± 0.07	1.69 ± 0.08	1.64 ± 0.05	1.61 ± 0.08	<0.001 *
BMI (kg/cm^2^)		26.01 ± 4.52	24.34 ± 3.32	26.87 ± 3.49	27.92 ± 3.65	0.003 †
Chronicity (months)		0	15.37 ± 37.13	88.32 ± 55.62	190.08 ± 160.20	<0.001 †
Sex	male	12 (48%)	13 (52%)	7 (28%)	4 (16%)	0.025 ‡
	female	13 (52%)	12 (48%)	18 (72%)	21 (84%)	

Abbreviations: BMI: body mass index; CI: confidence interval; HV: hallux valgus; SD: standard deviation. * Mean ± SD and one-way analysis of variance (ANOVA) were used. † Mean ± SD and the Kruskal–Wallis test were used. ‡ Frequency, percentage (%), and the Chi-squared test (χ^2^) were utilized. In all analyses, *p* < 0.05 (with a 95% CI) was considered statistically significant.

**Table 2:** Comparisons of outcome measurement scores among different HV deformity degrees.

Outcome Measurements	No HV Degree-I (n = 25)	Mild HV Degree-II (n = 25)	Moderate HV Degree-III (n = 25)	Severe HV Degree-IV (n = 25)	*p*-Value (η^2^)	Bonferroni *p*-Value (*d*) (1) I vs. II (2) I vs. III (3) I vs. IV (4) II vs. III (5) II vs. IV (6) III vs. IV
Kinesiophobia total score (TSK-11)	19.64 ± 4.81	21.60 ± 6.07	23.64 ± 4.56	26.36 ± 4.49	<0.001* (η^2^ = 0.203)	(1) (*d* = 0.35) (2) (*d* = 0.85) (3) <0.001 (*d* = 1.44) (4) 0.928 (*d* = 0.38) (5) (*d* = 0.89) (6) 0.353 (*d* = 0.60)
Activity avoidance score (TSK-11)	12.84 ± 3.11	13.76 ± 4.56	15.44 ± 3.96	17.24 ± 3.41	<0.001 † (η^2^ = 0.168)	(1) (*d* = 0.23) (2) 0.127 (*d* = 0.87) (3) (*d* = 1.34) (4) 0.392 (*d* = 0.39) (5) (*d* = 0.86) (6) 0.851 (*d* = 0.48)
Harm score (TSK-11)	6.80 ± 2.29	7.84 ± 2.19	8.20 ± 2.16	9.12 ± 2.02	0.002 † (η^2^ = 0.132)	(1) 0.599 (*d* = 0.46) (2) 0.205 (*d* = 0.62) (3) (*d* = 1.07) (4) (*d* = 0.16) (5) 0.177 (*d* = 0.60) (6) 0.530 (*d* = 0.43)
Pain intensity (NRS)	0	1.12 ± 1.26	6.40 ± 1.87	7.36 ± 1.55	<0.001 † (η^2^ = 0.850)	(1) 0.420 (*d* = N/A) (2) <0.001 (*d* = N/A) (3) <0.001 (*d* = N/A) (4) <0.001(*d* = 3.31) (5) <0.001 (*d* = 4.41) (6) 1.000 (*d* = 0.55)

Abbreviations: CI, confidence interval; d, Cohen d coefficient; HV, hallux valgus; η^2^, Eta-squared coefficient; N/A, not applicable; NRS, numeric rating scale; SD, standard deviation; TSK-11, Tampa Scale of Kinesiophobia–11 items. * Mean ± SD and one-way analysis of variance (ANOVA) completed with Bonferroni’s correction were used. † Mean ± SD and Kruskal-–allis test completed with Bonferroni’s correction were used. In all analyses, *p* < 0.05 (with a 95% CI) was considered statistically significant.

**Table 3:** Linear regression model for the kinesiophobia total score multivariate among HV deformity degrees.

Parameter	Model	*R*^2^ Change	Model *R*^2^
Kinesiophobia total score (TSK-11)	15.136		
	+0.101 * Age (years) +0.050 * HV deformity degrees	0.271 ‡ 0.029 †	0.300

Abbreviations: HV, hallux valgus; TSK-11, Tampa Scale of Kinesiophobia–11 items. * Multiplay: HV deformity degrees (I degree (no HV) = 1; II degree (mild HV) = 2; III degree (moderate HV) = 3; IV degree (severe HV) = 4). † *p*-value < 0.05 for a 95% confidence interval was shown, ‡ *p*-value < 0.001 for a 95% confidence interval was shown.

**Table 4:** Linear regression model for the pain intensity multivariate among HV deformity degrees.

Parameter	Model	*R*^2^ Change	Model *R*^2^
Pain intensity (NRS)	–3.993 +2.276 * HV deformity degrees +0.040 * Age (years)	0.776 ‡ 0.040 ‡	0.815

Abbreviations: HV: hallux valgus; NRS: numeric rating scale. * Multiplay: HV deformity degrees (I degree (no HV) = 1; II degree (mild HV) = 2; III degree (moderate HV) = 3; IV degree (severe HV) = 4). ‡ *p*-value < 0.001 for a 95% confidence interval was shown.
